# Methodological Approach to Designing Fermented Dairy Products with Optimal Biological Value

**DOI:** 10.3390/foods11010114

**Published:** 2022-01-03

**Authors:** Zinaida S. Zobkova, Ekaterina G. Lazareva, Vladislav K. Semipyatniy

**Affiliations:** All-Russian Research Institute of the Dairy Industry, Lusinovskaya Str. 35, 115093 Moscow, Russia; z_zobkova@vnimi.org (Z.S.Z.); e_lazareva@vnimi.org (E.G.L.)

**Keywords:** biotesting, *T. pyriformis*, fermented dairy products, relative biological value, methodology

## Abstract

The development of food products implies ensuring the optimal composition and ratio of the basic components, as well as their technological compatibility. A priori, the quality of raw materials, the optimal formula of the product and the efficiency of the technological process determine the quality of products, including biological value. The use of whole-cell sensors such as infusoria *Tetrahymena pyriformis* is most productive for screening biological studies. At present, for a comprehensive assessment there are no data on the use of simplest technology of fermented dairy products and the design of their biological value. The purpose of this research is to develop a methodology for creating whole-milk products of optimal biological value using the express method of biotesting. The research object was yogurt with the ratio of the mass fraction of fat and protein in the range of 0.36 ÷ 1.5, sucrose in the range of 5 ÷ 10%. An express method for determining the relative biological value of fermented dairy products using test organisms and an original methodology for creating whole-milk products of optimal biological value have been developed. A software has been developed to calculate formula of the product optimized for the following indicators: the relative biological value of the product, the cost of raw material and basic materials. The methodology is a tool to assist industry organizations in improving production technologies and quality management systems.

## 1. Introduction

One of the ways to solve the problem of population’s nutrition and health is to incorporate biologically valuable food products in a diet [1,2,3,4,5,6,7,8,9,10].

From the moment of receiving raw materials to the finished product at the stages of transportation, storage and processing, significant changes in nutrients occur due to microbiological, thermochemical, hydrolytic, oxidative and other processes, which significantly affect the quality of the product. In addition, the following factors create particular problems in ensuring the quality of food:

- Change in the composition of the raw material;
- Introduction of new ingredients;
- Creation of combined products;
- Improvement of traditional and development of new technologies for processing raw materials;
- Use of non-standard and secondary raw materials for food purposes.

There is a need for new methodologies for developing technologies and assessing their effectiveness and, consequently, the usefulness of the product. It is necessary to select technological modes that ensure the production of a finished product with a high indicator of its biological value. A biological assessment will show that for a dairy product there are boundaries of technological impact which, when exceeded, lead to a decrease in the biological value of products obtained from animal raw materials rather than an increase [11].

Traditional microbiological and physicochemical methods of controlling raw materials and finished products do not allow assessing all factors that affect not only their quality, but also the quality of product consumer’s life in general.

The most reliable method for assessing the quality of products from this point of view is the biological method using higher animals [12,13]. However, its application is difficult for regular product control, evaluation of raw materials, various processing methods and new technologies. For the biological assessment of raw materials and finished products, the simplest life forms can be used as indicators, the main parameters of metabolic processes of which are close to those of higher organisms [14,15,16,17].

The advantages of using accelerated methods of biological assessment using the simplest test organisms are that they are quite simple, cheap and highly sensitive, they can drastically reduce the research time, the amount of material required and can assess the quality of various substrates, including those that are of little use for analysis in higher animals (such as liquid ones, those low in protein, etc.). In addition, protozoan bioassay is a more humane and cost-effective alternative to vertebrate animal research.

Analysis of data in literature and previous studies indicate that among the currently existing test organisms Tetrahimena piriformis ciliates most closely correspond to higher organisms in terms of their physiological and biochemical parameters and toxic and biological reactions to the effects of certain substances.

To obtain objective and reliable information about the biological value of objects, Dolgov V.A. [16,17,18,19] and other researchers proposed to use a set of various manifestations of the vital activity of tetrachimens, which includes the survival of protozoa, their behavioral characteristics, growth response, coefficients: efficiency and use of protein, nitrogen metabolism, biological activity, biological potential, dye-binding capacity of cells, biochemical and morphological parameters. The proposed appropriate methodological techniques can significantly increase the information content of test functions and facilitate their registration, use in the developed accelerated methods for determining the harmlessness and biological value of feed, food and environmental objects.

The above set of indicators characterizes the entire life cycle of ciliates as fully as possible, reflects the presence of an adaptation period, its nature, the urgent and long-term influence of the tested substances or products on various aspects of life and the morphology of protozoa, which makes it possible to assess the toxicity and/or biological value of the tested objects. However, when using the express method for utilitarian purposes, its optimization is required, taking into account the fact that some of the above characteristics are interdependent.

The purpose of the research is to develop a methodology for creating whole-milk products of optimal biological value based on the use of the express method of biotesting.

## 2. Materials and Methods

The research was conducted in the Laboratory of New Milk Products Technologies of the All-Russian Scientific Research Institute of the Dairy Industry (VNIMI) together with the Laboratory of Veterinary and Sanitary Expertise of the All-Russian Research Institute of Experimental Veterinary Medicine named after K.I. Scriabin and Y.R. Kovalenko of the Russian Academy of Sciences (VNIIVSGE).

The research objects were as follows: a normalized milk mixture and a fermented milk product obtained taking into account technological limitations (the ratio of the mass fraction of fat and protein; the mass fraction of sucrose; the mode of thermal treatment; the types of microorganisms used as starter cultures) and using functional ingredients.

The methodology was developed using yogurt with added sugar and functional ingredients—stabilizing additives and transglutaminase as an example.

### 2.1. Production of Yogurt for Research

The experiment was carried out on the raw material of one batch of the same supplier (whole milk powder, Meleuzovsky Milk Canning Plant, Russian Federation; batch—86; production date—22 March 2020; mass fraction of fat—26.1%; protein—27%; dry matter—97.1%; carbohydrates—38%). Whole milk powder was reconstituted, separated and normalised mixtures were made considering the mass fractions of protein, fat, skimmed milk solids and sucrose. Selected stabilizers were added to the mixture in dry form. Transglutaminase (TG) was added into normalized milk at a dose of 0.05% in dry form and it was kept at a temperature of (4 ± 2) °C for 18 h.

Normalized milk mixtures were heated to a temperature of (65 ± 2) °C and homogenized with the laboratory valve homogenizer Manton-Gaulin (Gaulin Corp, Boston, MA, USA) at a pressure of 17 ± 2.5 MPa. The homogenized mixture was pasteurized at a temperature of 92 ± 2 °C with an exposure time of 10 min. Further, the mixture was cooled down to the fermentation temperature and it was fermented. For the fermentation of yogurt, strains of starter microorganisms (*Str. thermophilus*, strains 6 kb and *Lactobacillus delbrueckii subsp. bulgaricus*, strain L37/1 (ratio 4:1) from the collection of the Laboratory of Microbiology of VNIMI were used. The normalized mixtures were fermented at 40 ± 2 °C for 4 h to an acidity of ≥75 °T (pH 4.5–4.6). The obtained fermented milk clot was partially cooled down to 25–30 °C and mixed. Further, the products were packaged in consumer packaging, hermetically sealed and sent for post-cooling, structure formatting for 16 h and storing in a refrigerator at a temperature of 4 ± 2 °C. The pH of the finished product was 4.2–4.3.

### 2.2. Assessment of the Relative Biological Value of Yogurt Using the Express Method of Biotesting 

The museum strain of VNIIVSGE *Tetrahymena pyriformis* (strain Wh-18) was used as a test organism. During testing the samples of low-fat and the optimal chemical composition yogurt (given below) made without the use of stabilizers and TG, served as control. Samples of the products were diluted with distilled water in various ratios. A total of 1 mL of diluted samples were mixed with 1 mL of carbohydrate-salt yeast medium (CSY) (1.5 g of glucose, 0.1 g of yeast extract, 0.1 g of sodium chloride, distilled water (pH of prepared samples—7.1) to 100 mL). Test tubes with prepared samples were closed with corks, placed in a boiling water bath on a tripod for 15–20 min to inactivate the microflora. After cooling to 5 °C, 0.02 mL of a 3-day-old culture of tetrahymena grown on a peptone medium was added to each tube. Cultivation of infusoria was carried out in a thermostat at 25 ± 2 °C, periodically shaking 2–3 times a day, for 4 days before the onset of the stationary growth phase. After 96 h, one drop of a 5% alcohol solution of iodine was added to the test tubes, thoroughly shaken and the grown cells were counted.

The number of cells was counted using a microscope in 10 large squares of the Fuchs-Rosenthal counting chamber; the size of the infusoria cells—in fixed smears using an eyepiece micrometer; the daily increase in biomass—by counting the number of living forms that grew on the first to fourth days of observation. The relative biological value (RBV) was determined by the ratio of the number of cells grown on the testing product to the number of infusoria grown on the control product, expressed as a percentage.

Comparative analysis of products with different chemical compositions was carried out under the condition of the same protein content in the medium. Since the samples under research differed significantly in fat content, the optimal dilution was selected to improve the comparability of the results. Milk with a different mass fraction of fat was studied in dilutions that provide the content of milk protein in the medium all expressed as N: 0.05, 0.15 and 0.3 mg/mL. In the analysis the samples used for comparison were the ones containing skimmed pasteurized milk. It was determined that the dilution of milk for testing to 0.15 mg of N/mL provided a sufficiently balanced intake of protein and fat by the infusoria, which allowed us to identify the effect of their ratio on the growth of test-organisms and determine its optimum.

### 2.3. Assessment of Physicochemical and Rheological Characteristics of Yogurt 

The chemical composition of the normalized mixture of finished product was determined by standardized methods: the fat—Gerber’s butyrometer method (ISO 2446:2008); the total protein—by Kjeldahl method with use of KJELTEC automatic system (ISO 8968-1:2014); the carbohydrate—by IDF 028A:1974.

The shear structural and mechanical characteristics (SMC) of yogurt were determined by rotational viscometer using a Brookfield viscometer (Brookfield Engineering Labs., Inc. model DV-II + Pro, Middleboro, MA, USA) with concentric cylinders (spindle SC4-21) at a temperature of 20 ± 2 °C. During the measurements, the range of change of the shear rate ($\dot{\gamma }$): 0.093 ÷ 9.3 $1{\;s}^{-1}$ was chosen, in which the product’s flow curves follow the pattern described in Ostwald-de Valle equation (power law) for a pseudoplastic medium:

(1) $$\eta \mathrm{eff}=K\;(\dot{\gamma }/{\dot{\gamma }}_{1}\;)\;n-1$$

where ${\dot{\gamma }}_{1}$—flow consistency index, equal $1{\;s}^{-1}$, K—parameter of effective viscosity at a single value of the shear rate (consistency parameter), n—flow index, a characterizing angle of inclination of the flow in logarithmic scales.

The moisture-retaining capacity (W, vol.%) was determined by centrifugation method of 10 mL of the destroyed clot (at a temperature of (20 ± 2) °C) for 30 min with a separation factor of F = 750. The result was calculated from the following:

W = 100 (10 − V)/10(2)

where V—volume of the released serum from 10 mL of the clot.

To compare the results, experimental and control samples were produced from the same batch of raw materials. Repetition of experiments—3 times. Observation results containing extreme outliers were excluded from consideration. The obtained experimental data were processed by methods of mathematical statistics using Microsoft Excel, Statistica, and DataFit programs. Where applicable statistical significance level is set to 0.05, and relative error of measurement is ±5%.

In order to improve the reservoir method for the production of fermented milk drinks, food additives with stabilizing properties are applied. Mixtures of various thickeners and gelling agents as well as the protein-crosslinking enzyme transgutaminase are suitable for this purpose.

### 2.4. The Choice of Stabilizers for the Study of Their Effect on the Yogurt RBV

For the research of the effect of stabilizers on the RBV of yogurt, the following commercial products from various manufacturers were selected: transglutaminase (TG) for milk powder (activity 120–130 u/g, SHEF technology company, Russian Federation), recommended dose 0.035%; low-esterified amidated pectin GRINDSTED^®^ Pectin LA 410 (E440), Danisco CJSC, recommended dose 0.1–0.15%; stabilizing systems for fermented milk products: GRINDSTED^®^ SB 550 A (E1442, E440), Danisco CJSC, recommended dose 0.9–1.3%; Stabisol JT 11 (gelatin, E1422, E440), recommended dose 0.5–0.9%; Stabisol Y5 (gelatin, E412), Hydrosol GmbH&Co, Germany, recommended dose 0.3–0.6%; Milkpro KM 23 (E1442, E1422, E412, gelatin), TU 10.89.19-002-12061283-2018, TK Milkpro Ural, recommended dose 0.4–13%. 

### 2.5. Methodology for Creating (Designing) Whole-Milk Products with Optimal Relative Biological Value

**Figure 1:**
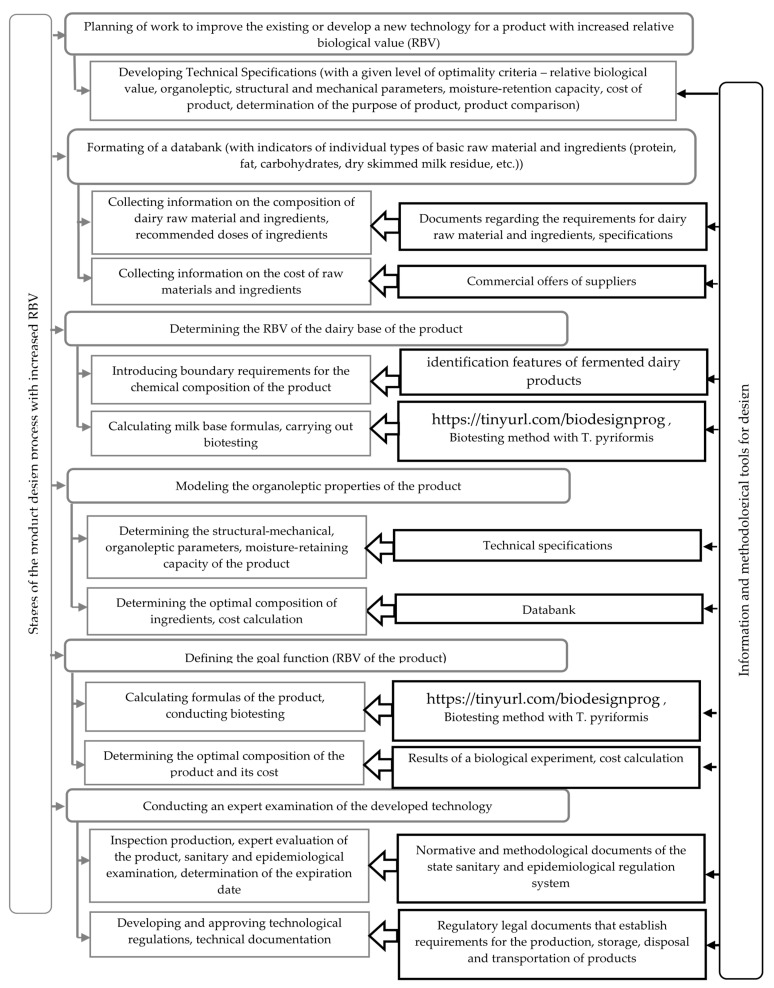
Algorithm for designing fermented dairy products with increased RBV.

The algorithm for solving the optimization problem of research is presented schematically in Figure 1.

At the first stage, an information database was designed, which included a list of the main raw material and ingredients, their chemical composition, functional properties and prices.

At the second stage, the restrictions on the chemical composition, organoleptic parameters were determined in accordance with the current standards and requirements for similar products, as well as RBV studies of milk base of the product.

Using the express method of biotesting with *Tetrahymena pyriformis*, the influence of the type of thermal treatment of milk (sterilization; UHT treatment; pasteurization; prolonged pasteurization for 3–4 h at 95–99 °C) was studied. The influence of the species composition of starter cultures [*Lc. lactis*; *Str. thermophilus*; *Lc. lactis subsp. lactis biovar diacetylactis*, *Acetobacter subsp. aceti*, *Propionibacterium freudenreichii subsp. shermanii*; *Str. thermophilus, Lactobacillus delbrueckii subsp. bulgaricus* (4:1 ratio)] was also investigated on RBV fermented dairy products. It was found that among the studied restrictions, the most significant is the type of starter cultures (the range of the RBV variation was 24%). The type of thermal treatment did not have a significant effect on the RBV of milk (the range of variation of 7% did not exceed the maximum error value).

In order to assess the effect of the mass fractions of fat, protein and sucrose in normalized milk a full-factor experiment was conducted on the RBV of yogurt. The control sample was yogurt made from skimmed milk.

At the third stage, the doses of the stabilizing additives selected for the analysis were determined, which provide the best performance of the yogurt intended for packaging in cups. For this purpose, yogurt was produced with an optimal chemical composition (mass fraction of fat 3.1%, protein 2.8%, sucrose 7.5%), TG and stabilizers added in various doses. In the product samples, the moisture-retaining capacity, SMC and organoleptic parameters were determined according to the developed five score scale (Table 1).

At the fourth stage, samples of yogurt with optimal chemical composition with stabilizers were tested on test organisms *T. pyriformis*.

At the fifth stage, a databank is designed which characterizes the properties of the product:

- The formula of the designed product with the optimal relative biological value is determined;
- The justification of the number of added ingredients that allow producing a product with the desired properties is given.

The final balanced linear equations are created: according to the chemical composition of the final product (according to the content of fat, protein, dry skimmed milk residue, carbohydrates, functional components) the cost of the main raw and other materials is determined, Russian ruble/kg of the product.

To calculate the formula of the product with the optimal composition and optimal relative biological value, an applied computer program has been developed, which can be found under the link: https://tinyurl.com/biodesignprog (accessed on 22 October 2021).

To calculate the optimal formula of the product, the following parameters are entered into the program:

- The required values of the indicators of the chemical composition of the product, taking into account the restrictions stipulated by law and determined during the biotesting of the milk base of the product, quantity of additives, %;
- The type and quantity of additives determined when evaluating their functional properties;
- Indicators of the chemical composition of raw material and its cost.

Output data: optimal composition of milk base, %; cost of dairy raw material and other materials, Russian ruble/kg of product; expected RBV of the finished product with the selected additive, %.

The variants of the developed formula of the product are analyzed from various points of view (technological, organoleptic, biological, economic) and the one that most fully meets the goal is selected.

The final stage is the testing of the developed technology (optimized formulas of the product) in production conditions and its adjustment, if necessary. In order to confirm the modes and planned storage periods, a sanitary and epidemiological examination of the developed formulas of the product and technological modes is carried out. Technological regulations and technical documentation are prepared and approved.

## 3. Results

Table 2 shows the results of determining the RBV of the tested yogurt. The parameter of variation of the results of experiments conducted in three-fold repetition was from 1.1 to 18.3.

**Figure 2:**
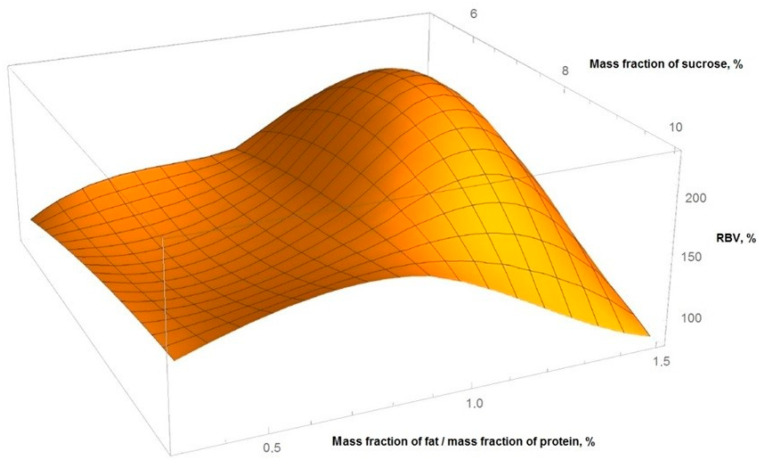
Graph of the RBV surface of yogurt for the variables of the mass fraction of fat, protein and sucrose.

Figure 2 shows the obtained graphical dependences of yogurt RBV from the ratio of the mass fraction of fat protein and the mass fraction of sucrose.

The calculation was carried out using a full factorial quadratic model of the experiment, with the boundary values of the mass fraction of sucrose from 5% to 10%, the ratio of mass fraction of fat to mass fraction of protein from 0.36 to 1.5. The resulting interpolation equation:

z = −256.28 + 65.252 x − 3.336 x² + 410.72 y − 13.081 x y − 174.84 y²

where: x—mass fraction of sucrose, y—ratio of mass fraction of fat to mass fraction of protein, z—RBV of yogurt.

The equivalence of the experiment plan was checked by the Cochran’s test, adequacy—using the Fisher’s test, the significance level in all places was assumed to be 0.05.

From the data obtained, it followed that the optimal ratio of the mass fractions of fat and protein in yogurt is in the range of 0.8–1.2%, sucrose—7.5–8.2%. At the same time, the maximum value of RBV was >180%.

**Figure 3:**
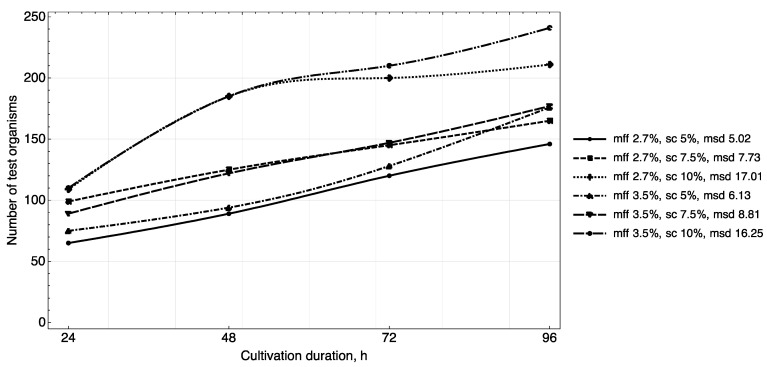
Growth dynamics of *T. pyriformis* in a medium with yogurt of different chemical composition (mff—mass fraction of fat, sc—percentage of sucrose, msd—maximum standard deviance).

**Figure 4:**
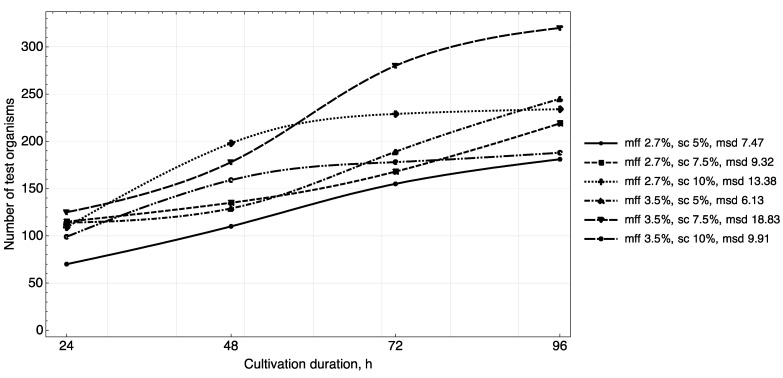
Growth dynamics of *T. pyriformis* in a medium with yogurt of different chemical composition (mff—mass fraction of fat, sc—percentage of sucrose, msd—maximum standard deviance).

**Figure 5:**
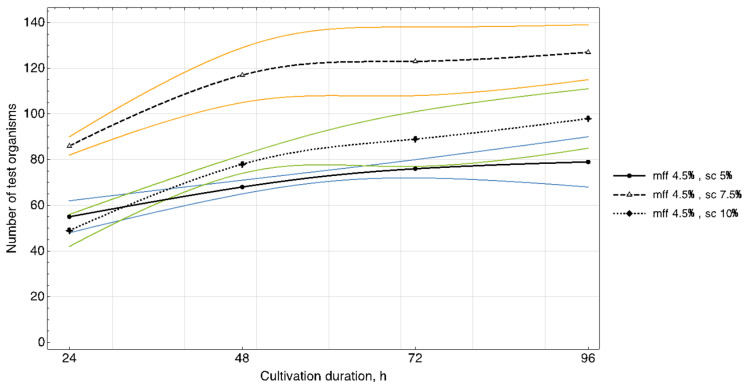
Growth dynamics of *T. pyriformis* in a medium with yogurt of different chemical composition with uncertainty bands (mff—mass fraction of fat, sc—percentage of sucrose).

The results of studying the daily increase in biomass in yogurt samples (Figure 3, Figure 4 and Figure 5) showed that in samples with a carbohydrate content of ~8.5% (mass fractions of sucrose 5.0%), the growth of *T. pyriformis* was uniform throughout all 4 days of observation.

The greatest increase in the test culture (increase in the number of cells by ~two times) was observed in the product with a carbohydrate content of ≥11% (mass fractions of sucrose 7.5% and 10%) in the first 3 days, then on the 4th day their growth relatively stabilized. In microscope slides of samples of yogurt with 10% content of sugar, a large number of lysed specimens and the presence of small tetrahymena (20–40 microns) of a spherical shape were observed, which indicates a large number of young specimens and acceleration of generations of test organisms. In the tested samples of yogurt containing 5% of sucrose, anatomical and morphological forms corresponding to adult tetrahymena specimens with a size of 50–60 microns prevailed. Judging by the dynamics of the growth of biomass and the morphology of tetrahymena, the content of 7.5% of sucrose in the product is most reasonable.

The fat content in the medium also influenced cell growth. The maximum increase in infusoria (222–374 units) was observed on the 4th day in the medium with yogurt with a fat content of 3.5%, 3.6% (mass fraction of protein content of 2.9%, 3.0%). A further increase of the fat content in the medium to 4.5% inhibited their growth (Figure 5).

Thus, the results of the study of the daily increase in biomass in yogurt samples and the morphological characteristics of test organisms corresponded to the trend identified during the full-factor experiment.

Table 3 shows the data obtained when comparing the results of instrumental and organoleptic evaluation of the consistency of yogurt with the optimal chemical composition.

The data are obtained from a statistical series of K values by dividing it into groups corresponding to each score and determining the value of the standard deviation of the K values, considering the measurement frequencies in each group.

From the data given in Table 3, the average reference value of the effective viscosity Kᵣ of yogurt at a temperature of (20 ± 2) °C, numerically equal to 9.8 Pa·s. The resulting average value can be used when selecting and determining the doses of additives that improve the consistency.

Table 4 shows the values of the effective viscosity of yogurt samples with TG and the studied stabilizers. The added doses of stabilizers provide the maximum organoleptic assessment and indicators of moisture-retaining capacity on the first and last day of storage.

**Figure 6:**
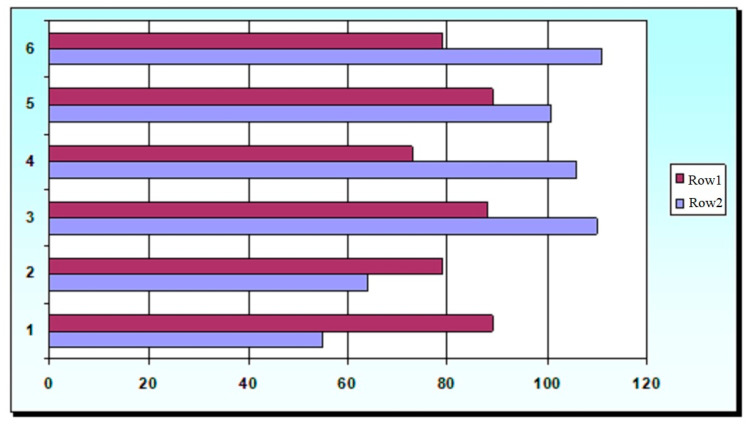
RBV (%, Row 2) and relative viscosity (%, Row 1) of yogurt samples made with stabilizers and TG: 1-Milkpro 23 KM; 2-TG; 3—GRINDSTED^®^ Pectin LA 410; 4—Stabisol JT11; 5—GRINDSTED^®^ SB 550 A; 6—Stabisol Y5.

Figure 6 shows the indicators of RBV and relative viscosity, expressed in % K from the reference value of Kᵣ = 9.8 Pa·s of yogurt samples made with stabilizers and TG.

## 4. Discussion

Recently, several authors [20,21,22,23,24,25,26] have developed the concept of “whole-cell biosensors” (WCB) as an alternative to classical methods of biological assessment. Prokaryotic or eukaryotic cells are used as biosensors. Previous studies have shown that eukaryotic cells of ciliates are similar to higher organisms in terms of the genome and several basic metabolic parameters [27,28], which allows interspecies extrapolation of the results of assessing the biological value of food products [19,29,30].

To determine the harmlessness (toxicity) and biological value of products, a modern, more convenient, and cheaper method for determining RBV on the simplest *Tetrahymena pyriformis* has been used recently as a test object is recognized by the world scientific community and is in demand.

Biotesting of food products for *T. pyriformis* requires individualization of methodological approaches to assessing the safety and biological value of products of different groups. Important methodological points during testing are the choice of the concentration of the test product, the composition of the *T. pyriformis* cultivation medium based on it, and the composition of the reference standard [31].

According to the method of biological assessment of the nutritional value of products using *T. pyriformis* by Stott and Smith, the test material was suspensions with a content of 5 mg of nitrogen in 1 mL of which dilutions with 2–5 mg of nitrogen in 1 mg were prepared. The nutrient medium was made up of specific proportions of 7 solutions of various vitamins, minerals, amino acids, glucose. Distilled water (up to 100 mL) was added, and the pH was adjusted with NaOH to 7.1. After sterilization at 121 °C for 10 min, the media were poured into 10 mL test vials with caps, inoculated with three drops of a 3-day broth culture of *T. pyriformis* W, and inoculated at 25 °C for 4 days in an inclined position (15°) for better aeration and rapid growth of the culture. After 4 days of incubation, 1 mL of a preservative liquid, consisting of 90 mL of water, 20 mL of 36% (*w*/*v*) formaldehyde, and 10 mL of a 3.5% solution of KH_2_PO_4_ + K_2_HPO_4_, was added to 1 mL of the culture medium, and the organisms were counted in a hemocytometer (depth 0–2 mm) according to Fuchs-Rosenthal in eight squares with an area of 1 mm. An average amount of 1 mm^2^ gave a final test culture population of $10^{4}$ organisms/mL.

For amino acid nutritional analysis, test materials were measured relative to a pure amino acid response curve and calculated as the average of the four nitrogen levels used. Values differing by more than 20% from the mean deviated. Statistical analysis of four separate experiments to evaluate amino acids gave coefficients of variation ranging from 7.6 to 14.4%.

Ignatiev A.D. et al. [32] proposed a modification of the method for biological assessment of the nutritional value of products using *T. pyriformis* by Stott and Smith. In particular, when analyzing milk, it is poured into sterile dark glass vials, kept in a water bath at 30 °C for 5–10 min, and thoroughly mixed. Use the essential medium for analysis: glucose—1.5 g, yeast extract—0.1 g, sea salt—0.1 g, distilled water—up to 100 mL (pH up to 7.1). The test samples are added to the vials with the main medium for analysis (1 mL) at the rate of 0.3 mg of total nitrogen per 1 mL of the medium. To obtain more representative results, it is proposed to use three weighed portions per sample (with the minimum, optimal, and maximum nitrogen content—0.1, 0.3, and 0.5 mg), and then average the data obtained. The vials are sterilized at 1 atm for 30 min or boiled for 15 min to inactivate the microflora. Each sample is tested in triplicate. After cooling the vials to room temperature, 0.02 mL of a three-day culture of ciliates, grown at 25 °C on a peptone medium of the following composition: bacteriological peptone—2 g, yeast extract—0.1 g, glucose—0.5 g, sodium chloride—0.1 g, distilled water—up to 100 mL (pH 7.1). The cultivation of ciliates is carried out for 4 days at 25 °C. For aeration, the samples are shaken at regular intervals 2–3 times a day. To calculate the number of grown ciliates, 3 mL of fixing solution (20 mL of 36% formalin solution, 175 mg of potassium dihydrogen phosphate, 170 mg of potassium hydrogen phosphate, 440 mL of distilled water) are added to the vials, shaken thoroughly. Then the cells of the ciliates are counted.

Zobkova Z.S. [33] determined the biological value of fermented milk drinks stored for a long time, based on the methodological recommendations of the All-Union Agricultural Academy [34,35,36], the following method was applied.

A total of 1–3 g of a fermented milk drink, thoroughly grind in a porcelain mortar so that the infusoria can swallow the particles of the drink. Samples are placed in antibiotic vials, 1 mL of a solution consisting of 0.1% yeast extract, 1.5% glucose, 0.1% sea salt in distilled water (pH 7.1) is added to them, inoculated with 0, 02 mL of a 3-day culture of *T. pyriformis* grown in the above peptone medium and incubated. Then add 3 mL of fixing solution to the vials and shake thoroughly. A drop of suspension is placed in a Fuchs-Rosenthal counting chamber, and ciliates are counted in 10 squares.

Bondaruk A.M. et al. [37,38] developed methodological approaches to assessing the safety and biological value of food products on *T. pyriformis*. The ranking of food products (meat, dairy, fats, grain, and products of its processing, fruits, and vegetables, potatoes) by nutritional composition and energy value has been done. Tables for calculating the number of food products for cultivation with *T. pyriformis*, corresponding to various levels of daily consumption of food and biologically active substances by humans, were compiled to extrapolate the results.

It was developed and experimentally tested two versions of the environment required to study the biological value and safety of food on *T. pyriformis*. The growth of *T. pyriformis* was studied in cultivation media containing human serum albumin, milk protein coprecipitate, and casein peptone as a protein component in addition to casein. The carbohydrate component of the cultivation medium was glucose, starch, and maltodextrin. The source of vitamins was yeast extract or a mixture of vitamins. Mineral elements were also added to the cultivation medium. Population growth was studied at a protein concentration in the culture medium of 1, 2, 4 mg/mL. The population size of ciliates was determined after 24, 48, 72, 96 h. It is proposed to use two comparison standards. Composition of standard No. 1: protein (casein, coprecipitate, peptone), carbohydrates (glucose, starch, maltodextrin), mineral elements, yeast extract. Composition of standard No. 2: protein (casein, coprecipitate, peptone), carbohydrates (glucose, starch, maltodextrin), mineral elements, vitamins. The choice of the standard depends on the composition of the test product and the purpose of the experiment. Therefore, standard No. 2 should be used for bio testing vitamin-containing specialized food products.

Bogdan A.S. et al. [39] biotesting of food grain of wheat, rye, and barley for *T. pyriformis*. The standard cultivation medium contained casein, glucose in an optimal ratio of 1:4, and the necessary set of minerals and vitamins. The initial value for calculating the indicators of the biological value of the product was the protein concentration, in which the population size in the stationary growth phase reached its maximum value. The protein concentration of 4 mg/mL, which ensures the maximum growth of *T. pyriformis* is a standard nutrient medium, was taken as the basis for calculating the biological value of cereals. The results of assessing the biological value of cereal food grains on *T. pyriformis* correlated well with the results of experiments on white rats and with calculations of amino acid rates.

In addition to the nutritional value of products, their safety is an essential factor. The *T. pyriformis* test organism can also be used for practical bioassay and product safety determination. The criterion for the toxicity of the object is a decrease in the increase in the number of ciliates in the test product relative to the increase in the number of ciliates in the control experiment. For example, Zobkova Z.S. et al. [40] studied cottage cheese by adding an enzyme preparation transglutaminase. The results showed that TG is not a toxic product.

As for the results of this research, lipids, which also contain essential substances, are of great importance in the formation of the degree of food usefulness. It is customary to evaluate the biological effect of lipids both by their digestibility, influence on the development of the body, and by a number of indicators of lipid metabolism.

The quantitative ratio of proteins and fats in the product composition affects the digestibility of both. When the fat content is too high, the separation of gastric juice is inhibited, the digestion of proteins by the digestive enzymes pepsin and trypsin slows down, the course of metabolism of certain substances changes, and the blood coagulation system and the process of assimilation of vitamins are suppressed.

Before the start of the research, there were no data from biomedical experiments on the optimal ratio of fat and protein in dairy products that provide maximum biological value.

When conducting tests, important methodological points are: the choice of the concentrations of the test product, the composition of the *T. pyriformis* cultivation medium based on it, and the composition of the reference sample

In the course of the research, factors influencing the relative biological value of fermented dairy products are revealed: the ratio of the mass fraction of fat and protein, the mass fraction of carbohydrates (sucrose), as well as functional technological ingredients: stabilizing additives, transglutaminase (yogurt as an example). The optimal value of the ratio of mass fraction of fat and protein in 0.8–1.2% yogurt, mass fraction of sucrose—7.5–8.2% was determined. At the same time, the maximum value of RBV was more than 180%.

From the data obtained, it follows that the presence of stabilizers in yogurt, which are mainly dietary fibers, and the pre-processing of milk with TG obviously somewhat reduced the bioavailability of nutrients. The presence of stabilizers in yogurt and pre-treatment of milk with TG led to a decrease in the growth rate of test organisms in some samples (RBV significantly decreased by 11–27%). No negative influence of the tested samples on the mobility, movement pattern, behavioral response, morphological parameters that cause toxic effects on *T. pyriformis* was found.

When comparing the values of the above indicators, it is obvious that the best functional properties (relative viscosity 101–110%, moisture-retaining capacity 96–100%) and at the same time the least effect on the RBV of the product (88, 89%) had Stabisol Y5, Milkpro KM 23.

Adequate biological tests were determined during the research on the relative biological value of the product. Based on the results obtained, an express method for determining the relative biological value of fermented dairy products using test organisms was developed.

The method for determining the relative biological value of dairy products using *Tetrahymena pyriformis* is based on establishing a difference (in %) between the number of ciliates that have grown in the analyzed sample (experiment) and the number of ciliates that have grown in the control sample (control) of the product.

## 5. Conclusions

Based on the express method of assessing the relative biological value and the selected criteria, a method for creating fermented milk products to ensure their quality and usefulness was developed using the example of yogurt. In addition, a program has been designed that allows calculating the formula of yogurt with a different composition of raw materials and a fat content from 0.5 to 10%, optimized according to the following indicators: the relative biological value of the product; the cost of raw materials and functional components. It can be assumed that *Tetrahymena pyriformis* can be recommended as a test organism suitable both for the biological evaluation of fermented milk products and for studying their safety.

## Figures and Tables

**Table 1:** Scoring scale of organoleptic evaluation of yogurt consistency.

Consistency Characteristics	Score
The surface is glossy, without separating the serum; the consistency is uniform, smooth, moderately viscous, dense, creamy	5
The surface is glossy, there is a slight separation of the serum; the consistency is not smooth enough, inhomogeneity is allowed, which disappears after intensive mixing, rather viscous, loose or somewhat excessively dense, jelly-like	4
Insufficiently viscous or excessively viscous, excessively dense consistency; rough, insignificant, single grain; the presence of serum on the surface	3
The consistency is non-uniform, coarse, with the separation of serum, liquid or excessively dense, heavy, gelled	2
The consistency is pronounced heterogeneous, flaky with a significant separation of serum, very liquid or mushy, coarse, gelled	1

**Table 2:** RBV of yogurt with different mass fractions of fat, protein and sucrose.

№ of Samples	Mass Fraction of Sucrose, % (x)	Ratio of Mass Fraction of Fat: Mass Fraction of Protein (y)	The Average Number of Cells in the Five Squares of the Counting Chamber, Units (z) for 3 Experiments	Standard Off, σ (Standard Error)	Parameter of Variation, CV = σ·100/z	RBV, % (Cell Growth Relative to Control)
1	2	3	4	5	6	7
K	0	0.02	132	8.2	6.2	100
1	5	0.36	146	10	6.8	111
2	5	0.69	176	10.97	6.2	133
3	5	0.89	181	9.7	5.36	137
4	5	1.2	245	21.7	8.8	186
5	5	1.5	104	16	15.4	79
6	7.5	0.36	165	18.2	11	125
7	7.5	0.69	177	6.8	3.8	134
8	7.5	0.89	219	8.08	3.7	166
9	7.5	1.2	320	47.1	14.7	242
10	7.5	1.5	160	10	6.3	121
11	10	0.36	211	13.5	6.4	160
12	10	0.69	236	2.5	1.1	179
13	10	0.89	234	5	2.1	177
14	10	1.2	188	35.6	18.3	142
15	10	1.5	109	11	10.1	83

**Table 3:** The organoleptic scoring scale and corresponding values of the effective viscosity of yogurt of the optimal chemical composition.

Organoleptic Scoring Scale	Effective Viscosity Range, K, Pa·s
3	3.4 ÷ 4.1; >13.6
4	5.3 ÷ 7,8; 12 ÷ 13.2
5	8.8 ÷ 10.8

**Table 4:** SMC, moisture-retaining capacity and consistency score of yogurt samples with stabilizing additives, TG and without them.

Stabilizing Additives (Doses)	Kᵢ *, Pa·s	Relative Viscosity, % (Kᵢ·100/Kᵣ)	W, %	Consistency Score
Control	2.5	-	76	-
TG (0.05%)	5.4	55	80	4
GRINDSTED^®^ Pectin LA 410 (0.15%)	6.3	64	90	4
Stabisol Y5 (0.6%)	10.8	110	100	4.5
GRINDSTED^®^ SB 550 A (1.3%)	10.4	106	94	5
Milkpro KM 23 (0.6%)	9.9	101	96	4.5
Stabisol JT11 (0.6%)	10.9	111	97	5

* value of the effective viscosity when the shear rate $\dot{\gamma }$ = 1 s^−1^.

## References

[1] Satterlee L.D., Kindrick J.G., Miller G.A. (1977). Rapid in vitro assay for estimating protein quality. Food Technol..

[2] Pellet P.L. (1978). Protein quality evaluation revisited. Food Technol..

[3] Pellett P.L., Young V.R. (1980). Nutritional Evaluation of Protein Foods.

[4] Vysotsky V.G., Yatsyshina T.A., Zilova I.S., Mamaeva E.M. (1980). The study of the biological value of proteins of various origins. Theor. Clin. Asp. Nutr. Sci..

[5] Vysotsky V.G., Zilova I.S. (1986). Medico-biological assessment of the quality and prospects for the use of proteins of some marine products. Bull. USSR Acad. Med Sci..

[6] Young V.R., Scrimshaw N.S., Bier D.M. (1981). Whole body protein and amino acid metabolism: Relation to protein quality evolution in human nutrition. J. Agric. Food Chem..

[7] Williams K., Sanders T. (2000). The relationship between health and consumption of protein, carbohydrates and fat. Nutr. Issues.

[8] Dotsenko V.A., Mosiychuk L.V. (2004). Diseases of Excessive and Insufficient Nutrition.

[9] Tutelyan V.A., Vyalkov A.I., Razumov A.N., Mikhailov V.I., Moskalenko K.A., Odinets A.G., Snezhneva V.G., Sergeev V.N. (2010). Scientific Foundations of Healthy Nutrition.

[10] Vysotsky V.G., Yatsyshina T.A., Rymarenko T.V., Mamaeva E.M. (1976). On methods for determining the biological value of proteins (review). Nutr. Issues.

[11] Vysotsky V.G., Shaternikov V.A. (1980). Adequacy for human definitions of the biological value of proteins by chemical and biological methods. Quest. Nutr..

[12] Evans E., Carruthers S. (1978). Comparisons of methods used for estimating the growth of Tetrahymena pyriformis. J. Sci. Food Agr..

[13] Lanokhina G.M., Irlina I.S., Etlin S.N. (1991). Features of the use of culture of infusoria in toxicological studies. Quest. Nutr..

[14] Dolgov V.A. (1992). Methodological Aspects and Practical Application of Accelerated Biological Assessment of Feed, Animal Products and Other Objects of Veterinary-Sanitary And Environmental Control. Ph.D. Thesis.

[15] Shemarova I.V. (2007). Methodological Recommendations on the Use of Tetrahymena Pyriformis GL Infusoria as Test Objects in Toxicological, Pharmacological and Environmental Studies.

[16] Dolgov V.A., Lapaev V.E. (1991). Methodological Recommendations The Use of Infusoria (Tetrachymena piriformis) as a Test Culture in the BioTester-2 Device (Express Method).

[17] Dolgov V.A., Lavina S.A. (2000). Methodological Recommendations for Accelerated Toxicological Assessment of Feed Using the BioTester-2 Device.

[18] Dolgov V.A., Lavina S.A., Arno T.S., Semenova E.A., Nikitchenko V.E. (2010). Methodical Manual on Biotest Assessment of Quality and Safety of Various Objects of Veterinary-Sanitary and Environmental Control.

[19] Dolgov V.A., Lavina S.A., Nikitchenko D.V. (2014). Assessment and interrelation of toxicity parameters of various substances for Tetrahymena pyriformis infusoria and white rats. Bull. RUDN.

[20] Belkin S. (2003). Microbial whole-cell sensing systems of environmental pollutants. Curr. Opin. Microbiol..

[21] D’Souza S.F. (2001). Microbial biosensors. Biosens. Bioelectron..

[22] Park M., Tsai S.-L., Chen W. (2013). Microbial biosensors: Engineered microorganisms as the sensing machinery. Sensors.

[23] Gutierrez J.C., Martın-Gonzalez A., Dıaz S., Ortega R. (2003). Ciliates as potential source of cellular and molecular biomarkers/biosensors for heavy metal pollution. Eur. J. Protistol..

[24] Gutierrez J.C., Martin-Gonzalez A., Diaz S., Amaro F., Ortega R., Gallego A., Lucas M.P. (2008). Ciliates as cellular tools to study the eukaryotic cell–heavy metal interactions. Heavy Metal Pollution.

[25] Zhu H., Tropsha A., Fourches D., Varnek A., Papa E., Gramatica P., Oberg T., Dao P., Cherkasov A., Tetko I.V. (2008). Combinatorial QSAR modeling of chemical toxicants tested against *Tetrahymena pyriformis*. J. Chem. Inf. Model..

[26] Shemarova I.V. (2012). Sensory Systems of Ciliates *Tetrahymena pyriformis* in Biotesting of Ecotoxicants and Biologically Active Substances. Ph.D. Thesis.

[27] Aury J.M., Jaillon O., Duret L., Noel B., Jubin C., Porcel B., Ségurens B., Daubin V., Anthouard V., Aiach N. (2006). Global trends of the whole-genome duplications revealed by the ciliate Paramecium tetraurelia. Nature.

[28] Eisen J.A., Coyne R.S., Wu M., Wu D., Thiagarajan M., Wortman J.R., Badger J.H., Ren Q., Amedeo P., Jones K.M. (2006). Macronuclear genome sequence of the ciliate Tetrahymena thermophila, a model eukaryote. PLoS Biol..

[29] Sinks G., Schultz T. (2001). Correlation of *Tetrahymena pyriformis* and Pimephales toxicity: Evaluation of 100 additional compounds. Environ. Toxicol. Chem..

[30] Rodionova L.G., Menshikova T.A., Dvoskin Y.G. (1999). Methodical manual. Alternative Research Methods (Express Methods) for the Toxic and Hygienic Assessment of Materials, Products and Environmental Objects.

[31] Bogdan A.S., Bondaruk A.M., Voitik N.P., Gusarevich N.V., Dubenetskaya M.M., Yenshina A.N., Zhukov A.M., Kedrova I.I., Kuznetsova Z.P., Likhoshva A.M. (1992). Studying the quality of crop production grown under conditions of intensification of production and technogenic pollution of the environment. Modern Methodology of Solving Scientific Problems of Hygiene.

[32] Ignatiev A.D., Shabliy V.Y.A. (1978). The Use of Tetrahymena pyriformis Infusoria as a Test Object in Biological Research in Agriculture.

[33] Zobkova Z.S. (1980). Improving the Technology of Fermented Milk Drinks in Order to Improve Their Quality and Increase Production Efficiency. Ph.D. Thesis.

[34] Belenky N.G., Ignatiev A.D., Shabliy V.Y., Isaev M.K., Nelyubin V.P., Boikov Y.I., Kharatyan S.G., Dolgov V.A. (1977). Methodological Recommendations for the Biological Evaluation of Animal Products and Feed Using the Tetrahymena Pyriformis Test Organism.

[35] Belenky N.G. (1990). The Use of the Express Method of Biological Evaluation of Products and Feeds.

[36] Belenky N.G. (1978). The biological value of animal products as the basis for choosing a rational technology for their production. Improving the Quality of Animal Products.

[37] Bogdan A.S., Bondaruk A.M., Tsygankov V.G., Kosyachenko G.E. (2013). Methodological approaches to the assessment of the biological value and harmlessness of food products on *Tetrahymena pyriformis*. Health and Environment: Collection of Scientific Works.

[38] Bondaruk A.M., Tsygankov V.G., Zhurikhina L.N., Fedorovich S.V., Gulin V.V. (2016). Assessment of the biological value and harmlessness of food products in order to develop rations for tourist and recreational activities. Proc. BSTU.

[39] Bogdan A.S., Bondaruk A.M., Zhurikhina L.N. (2010). The biological value of food grains of wheat, rye and barley according to the results of the evaluation of *Tetrahymena pyriformis*. Health Environ..

[40] Zobkova Z.S., Zenina D.V., Fursova T.P., Gavrilina A.D., Shelaginova I.R. (2015). Development of technologies for dairy products of healthy nutrition: Modern methodologies. Dairy Ind..

